# Monitoring an Alien Invasion: DNA Barcoding and the Identification of Lionfish and Their Prey on Coral Reefs of the Mexican Caribbean

**DOI:** 10.1371/journal.pone.0036636

**Published:** 2012-06-01

**Authors:** Martha Valdez-Moreno, Carolina Quintal-Lizama, Ricardo Gómez-Lozano, María del Carmen García-Rivas

**Affiliations:** 1 El Colegio de la Frontera Sur, Unidad Chetumal, Quintana Roo, Mexico; 2 Parque Nacional Arrecifes de Cozumel, Comisión Nacional de Áreas Naturales Protegidas, Chetumal, Quintana Roo, Mexico; 3 Reserva de la Bisofera Banco Chinchorro, Parque Nacional Arrecifes de Xcalak Comisión Nacional de Áreas Naturales Protegidas, Chetumal, Quintana Roo, Mexico; Smithsonian’s National Zoological Park, United States of America

## Abstract

**Background:**

In the Mexican Caribbean, the exotic lionfish *Pterois volitans* has become a species of great concern because of their predatory habits and rapid expansion onto the Mesoamerican coral reef, the second largest continuous reef system in the world. This is the first report of DNA identification of stomach contents of lionfish using the barcode of life reference database (BOLD).

**Methodology/Principal Findings:**

We confirm with barcoding that only *Pterois volitans* is apparently present in the Mexican Caribbean. We analyzed the stomach contents of 157 specimens of *P. volitans* from various locations in the region. Based on DNA matches in the Barcode of Life Database (BOLD) and GenBank, we identified fishes from five orders, 14 families, 22 genera and 34 species in the stomach contents. The families with the most species represented were Gobiidae and Apogonidae. Some prey taxa are commercially important species. Seven species were new records for the Mexican Caribbean: *Apogon mosavi, Coryphopterus venezuelae, C. thrix, C. tortugae*, *Lythrypnus minimus*, *Starksia langi and S. ocellata*. DNA matches, as well as the presence of intact lionfish in the stomach contents, indicate some degree of cannibalism, a behavior confirmed in this species by the first time. We obtained 45 distinct crustacean prey sequences, from which only 20 taxa could be identified from the BOLD and GenBank databases. The matches were primarily to Decapoda but only a single taxon could be identified to the species level, *Euphausia americana*.

**Conclusions/Significance:**

This technique proved to be an efficient and useful method, especially since prey species could be identified from partially-digested remains. The primary limitation is the lack of comprehensive coverage of potential prey species in the region in the BOLD and GenBank databases, especially among invertebrates.

## Introduction

Since the first appearance of the exotic lionfish in the western Atlantic [1], there has been great concern about the potential impact on coral reefs in the Caribbean region. A number of studies have recently been published on the lionfish invasion, in particular, the geographical distribution [2], the feeding behavior in the Bahamas [3], an analysis of cytochrome B mtDNA sequences to examine founder effects and for species identifications [4], establishing a molecular phylogeny [5], use of nursery habitats such as mangroves, [6], and evaluating native predator species [7].

Two species of lionfishes have been recorded as invaders in the western Atlantic: *Pterois volitans* (Linneo, 1758) and *Pterois miles* (Bennet, 1828). Although once considered to be synonyms, sequence differences in cytochrome b have confirmed the separation of the two species [8]. Nevertheless, despite cytochrome b is an important marker for species determination and it was successfully used to discriminate both, the barcodes, based in sequences of the cytochrome oxidase I, are becoming a wider standard in species identification (see www.fishbol.org).

At present, the lionfish invasion has spread to all along the coastal Yucatan Peninsula, including the entire Mesoamerican coral reef and has been recorded throughout the Caribbean as far as Venezuela [2], [9]. Recently, and for the first time, a larval lionfish was collected and reported in the Atlantic Ocean [10]. In the beginning, seem to this species was introduced as an ornamental fish, and later it escaped from an aquarium located in Florida [1], [2], [11].

**Table 1:** *Pterois volitans* COI sequences composition (from 30 samples).

Sequence composition (%)	Min	Mean	Max	SE
Guanine	18.96	19.75	20.19	0.028
Citocyne	26.66	26.98	27.01	0.009
Adenine	23.16	23.22	23.61	0.017
Tyrosine	29.7	30.06	30.41	0.013
Guanine-Citocyne	45.97	46.73	46.97	0.024
Guanine-Citocyne codon position 1	54.2	56.07	56.13	0.047
Guanine-Citocyne codon position 2	41.86	42.81	44.01	0.041
Guanine-Citocyne codon position 3	40.03	40.83	41.52	0.047

DNA barcodes have proven to be more than 90% successful in the identification of marine fish species in studies from Australia [12] and Mexico, where they also were used to connect developmental stages unidentified with adults [13]. One of the first and more important applications of this technique has been to detect exotic species in a fast, reliable and cost-effective way [14]. For example, exotic moths have been detected among field-caught populations [15] and an invasive microcrustacean, as the cladoceran Daphnia lumholtzi, has been discovered in Mexican freshwaters [16]. Another useful application of this method is the analysis of dietary habits. This approach has recently been used for an analysis of bat feces, since DNA barcoding permits the identification of prey in the absence of morphological evidence after digestion [17]. In case of fishes, two previous studies have used this technique, one to analyze herbivorous fish diets [18], and the other confirming the utility of the technique for piscivorous fishes, but in the laboratory [19].

**Table 2:** Lionfish (*Pterois volitans*) specimens collected in the different localities from Mexican Caribbean.

Locality	Specimenscollected	Specimens withstomach content	Collecting date(year)	Min-Max length ofthe specimens (mm)
Cozumel	58	47	2009	28–216
Xcalak	59	54	2009, 2010	40–262
Mahahual	35	21	2010	70–320
Isla Contoy	10	6	No data	10–90
Banco Chinchorro	13	11	2009	60–282
Puerto Morelos	1	1	2009	76–308
Playa del Carmen	2	1	2009	330
Isla Mujeres	9	3	No data	20–70

**Table 3:** List of fishes prey identified in the stomach contest of lionfish (*Pterois volitans*) by DNA barcoding analysis.

Order	Family	Genus	Species	No. of specimens	Similarity (%)
Beryciformes	Holocentridae	*Sargocentron*	*Sargocentron coruscum*	1	100
Perciformes	Apogonidae	*Apogon*	*Apogon lachneri*	2	100
Perciformes	Apogonidae	*Apogon*	*Apogon maculatus*	2	100
Perciformes	Apogonidae	*Apogon*	*Apogon mosavi* *	1	99.68
Perciformes	Apogonidae	*Apogon*	*Apogon townsendi*	2	100
Perciformes	Apogonidae	*Astrapogon*	No match found	1	
Perciformes	Apogonidae	*Astrapogon*	*Astrapogon puncticulatus*	1	99.84
Perciformes	Gobiidae	*Coryphopterus*	*Coryphopterus venezuelae* *	6	99.69
Perciformes	Gobiidae	*Coryphopterus*	*Coryphopterus eidolon*	2	100
Perciformes	Gobiidae	*Coryphopterus*	*Coryphopterus hyalinus*	2	100
Perciformes	Gobiidae	*Coryphopterus*	*Coryphopterus thrix* *	2	99.85
Perciformes	Gobiidae	*Coryphopterus*	*Coryphopterus tortugae* *	6	100
Perciformes	Gobiidae	*Priolepis*	*Priolepis hipoliti*	1	99.69
Perciformes	Gobiidae	*Lythrypnus* *	*Lythrypnus minimus* *	6	99
Perciformes	Grammatidae	*Gramma*	*Gramma loreto*	3	99.84
Perciformes	Haemulidae	*Haemulon*	*Haemulon flavolineatum*	3	100
Perciformes	Labridae	*Halichoeres*	*Halichoeres garnoti*	22	100
Perciformes	Labridae	*Thalassoma*	*Thalassoma bifasciatum*	11	100
Perciformes	Labrisomidae	*Malacoctenus*	*Malacoctenus triangulates*	2	99.69
Perciformes	Labrisomidae	*Starksia*	*Starksia ocellata* *	1	99.38
Perciformes	Labrisomidae	*Starksia*	*Starksia langi* *	1	99
Perciformes	Pomacentridae	*Abudefduf*	*Abudefduf saxatilis*	1	100
Perciformes	Pomacentridae	*Stegastes*	*Stegastes partitus*	6	99.85
Perciformes	Scaridae	*Scarus*	*Scarus iseri*	2	100
Perciformes	Scaridae	*Scarus*	*Scarus taeniopterus*	1	100
Perciformes	Scaridae	*Sparisoma*	*Sparisoma aurofrenatum*	8	100
Perciformes	Scaridae	*Sparisoma*	*Sparisoma viride*	2	100
Perciformes	Serranidae	*Cephalopholis*	*Cephalopholis cruentata*	3	100
Perciformes	Serranidae	*Liopropoma*	*Liopropoma rubre*	2	100
Perciformes	Tripterygiidae	*Enneanectes*	*Enneanectes altivelis*	3	100
Perciformes	Tripterygiidae	*Enneanectes*	*Enneanectes boehlkei*	1	100
Pleuronectiformes	Bothidae	*Bothus*	*Bothus lunatus*	1	100
Scorpaeniformes	Scorpaenidae	*Pterois*	*Pterois volitans*	16	100
Tetraodontiformes	Monacanthidae	*Monacanthus*	*Monacanthus tuckeri*	1	100

Also is showing percent of closest matches to reference sequences on BOLD.

* New range extension for Mexican Caribbean.

In this study, we apply the DNA barcoding method to analyze the prey composition for the carnivorous lionfish. The material studied comes from several collections of lionfish in Cozumel, along the Mexican portion of the Mesoamerican Coral Reef. Our primary goals were to establish, based on DNA barcodes, which species of Pterois is present on the Mexican Caribbean reef and which species comprise the diet of lionfish, based on the analysis of stomach contents.

**Figure 1:**
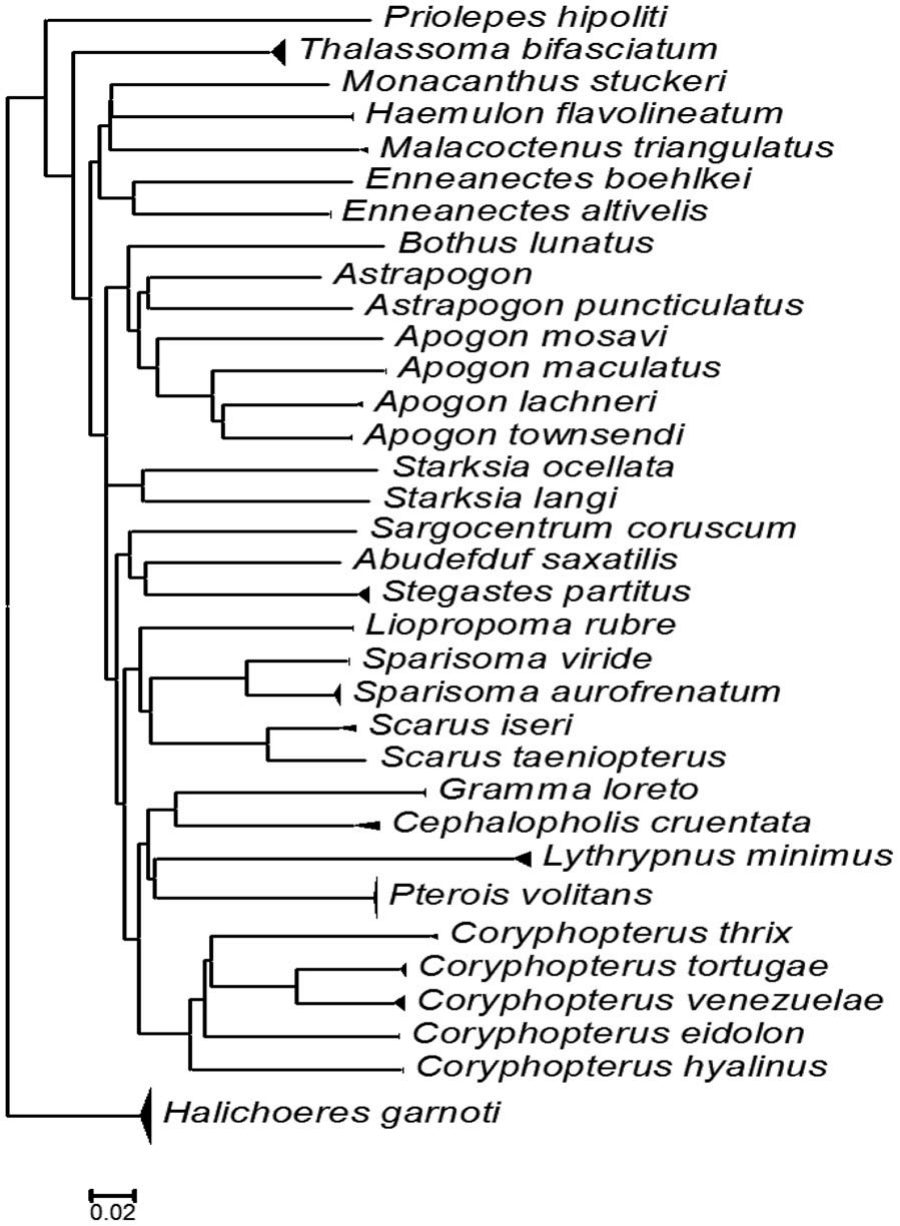
Neighbour joining tree for 34 fish species found in the stomach contents of the lionfish. Tree is based on genetic distances (K2P) for the COI gene; the base of the triangle gives a rough idea of the most consumed preys.

## Results

This study is the first report of the application of DNA barcoding to determine the prey composition for the invasive lionfish in the Atlantic Ocean. Partially-degraded biological material, such as stomach contents, can yield small PCR DNA fragments, sometimes less than 200 bp in length. Nevertheless, DNA barcoding can identify species with fragments as short as 100 bp with at least 90% efficiency [20]. The development of these mini-barcodes permits the species identification. This opens a great possibility to obtain sequences from short DNA fragments, quickly and cheaply [21].

### DNA Barcode Identification of Lionfish Adults

*Pterois volitans* and *P. miles* overlap in most morphological and meristic characters but do have different DNA sequences [8]. All sequences we obtained from 30 adult lionfish in the Mexican Caribbean matched with Indo-Pacific *Pterois volitans* with over 99% similarity. The average K2P distance among individuals was 0.054%.The mean sequence composition was guanine 19.75%, cytosine 26.98%, adenine 23.22%, tyrosine 30.06%, GC 46.73%. GC% Codon position 1, 56.07, GC% Codon position 2, 42.81 and GC% Codon position 3, 40.83 (Table 1).

**Table 4:** List of crustaceans prey identified in the stomach contest of lionfish (*Pterois volitans*) by DNA barcoding analysis.

Order	Family	Genus	Specie	Similarity (%)
Decapoda	Alpheidae	*Synalpheus*	*	99.24
Decapoda	Hyppolytidae	*Thor*	*	93.18
Decapoda	Palaemonidae	*	*	88
Decapoda	Palaemonidae	*	*	88.24
Decapoda	*	*	*	84.30
Decapoda	*	*	*	84
Decapoda	*	*	*	85.71
Decapoda	*	*	*	86.2
Decapoda	*	*	*	85.63
Decapoda	*	*	*	85.34
Decapoda	*	*	*	83.3
Decapoda	*	*	*	82.83
Decapoda	*	*	*	82.82
Decapoda	*	*	*	96.52
Decapoda	*	*	*	*
Decapoda	*	*	*	79.22
Euphausiacea	Euphausiidae	*Euphausia*	*Euphausia americana*	100
Stomatopoda	Gonodactylidae	*	*	87.2
Stomatopoda	Pseudosquillidae	*Pseudosquilla*	*	95.39

Also is showing percent of closest matches to reference sequences on BOLD.

* Unable to match with any records in BOLD database.

**Figure 2:**
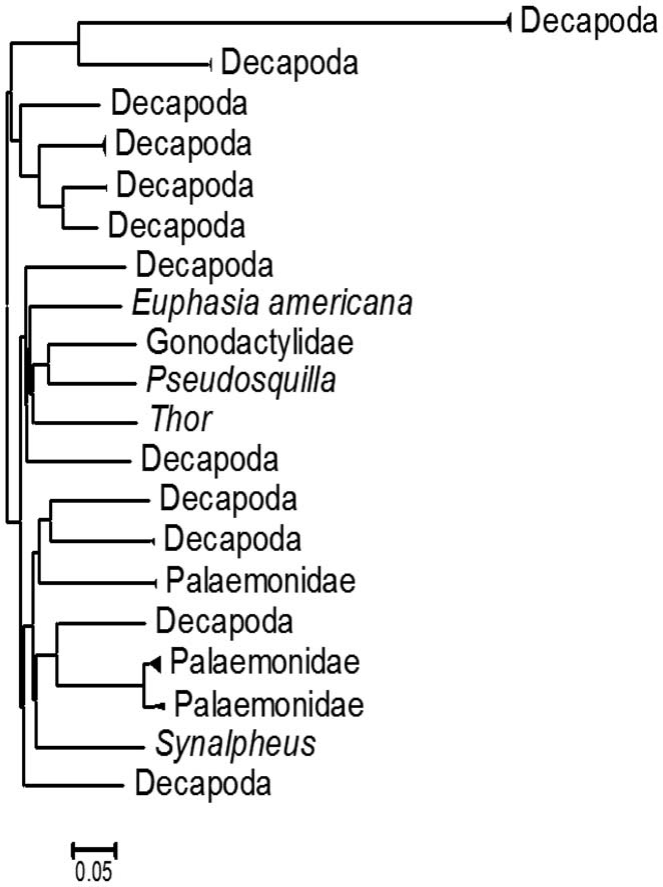
Neighbour joining tree for 20 clades representing crustaceans in the stomach contents of the lionfish. Each clade represents a different species, only one could be identified with no doubts; the base of the triangle gives a rough idea of the most consumed crustaceans.

### Identification of Prey Based in DNA Barcoding

Of the 157 stomachs examined, 144 had measurable contents (Table 2). In total 330 prey items were obtained but about 90% were mostly digested specimens. These included fish, typically only body parts or fragments of skeleton and tissue. As a result, most prey items were impossible to visually identify, even to order level. Some crustaceans were almost complete and could be identified before barcoding. All of the prey tissue fragments were barcoded, but only 168 yielded readable sequences. The read lengths in the majority (85%) were more than 600 bp long, while the remaining sequences had segments between 500 and 300 bp (mainly crustaceans) and only two sequences were less than 200 bp. There were no insertions, deletions or stop codons in any sequence. The sequences were compared to the reference library of sequences in the Barcode of Life Database (BOLD). Of the 168 sequences, 125 matched with fishes and 43 with crustaceans. In case of the fish sequences, 94% matched with greater than 99.38% similarity to reference sequences in BOLD, allowing identification to the species level. The remaining 6% could be identified only to genus (Table 3).

**Figure 3:**
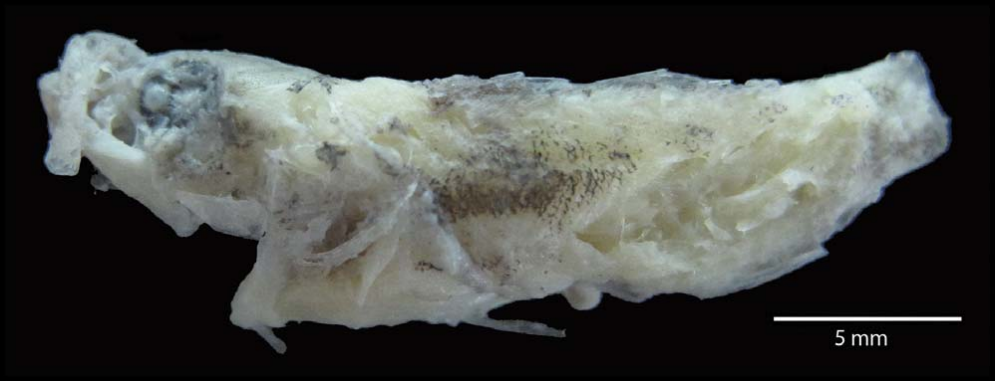
Specimen morphologically identifiable as a lionfish, from the stomach content.

Five orders of fishes comprising 14 families, 22 genera and 34 species were identified. The families with the greatest number of species were Gobiidae (7) and Apogonidae (6) followed by Scaridae (4), Labrisomidae (3), Labridae, Pomacentridae, Tripterygiidae, Serranidae (2), Holocentridae, Grammatidae, Haemulidae, Scorpaenidae, and Monacanthidae (1) (Figure 1, Table 3).

The total fishes species identified in the stomach contents (Table 3, Figure 1) include 27 species previously reported in the Mexican Caribbean: *Sargocentron coruscum, Apogon lachneri, A. maculatus, A. townsendi, Astrapogon puncticulatus, Coryphopterus eidolon, C. hyalinus, Priolepis hipoliti, Gramma loreto, Haemulon flavolineatum, Halichoeres garnoti, Thalassoma bifasciatum, Malacoctenus triangulatus, Abudefduf saxatilis, Stegastes partitus, Scarus iseri, S. taeniopterus, Sparisoma aurofrenatum, S. viride, Cephalopholis cruentata, Liopropoma rubre, Enneanectes altivelis, Enneanectes boehlkei, Bothus lunatus, Pterois volitans*, *Monacanthus tuckeri*, and seven species unreported before: *Apogon mosavi, Coryphopterus venezuelae, C. thrix, C. tortugae, Lythrypnus minimus, Starkia langi* and *S. ocellata.*

In terms of percent composition by number (%N) fishes dominated the lionfish diet (74.4%). The fish families with highest %N were Labridae (26.4%), comprising *Halichoeres garnoti* (17.6%) and *Thalassoma bifasciatum* (8.8%); Gobiidae (20%), comprising *Coryphopterus venezuelae* (4.8%), *C. tortugae* (4.8%) *Lythrypnus* (4.8%), *C. eilodon* (1.6%), *C. hyalinus* (1.6%), *C. thrix* (0.8%), and *Priolepis hipoliti* (0.8%); Scorpaenidae (12.8%) comprised the one species *Pterois volitans*; and Scaridae (10.4%) comprising *Sparisoma aurofrenatum* (6.4%) *Scarus iseri* (1.6%), *S. viride* (1.6%), and *S. taeniopterus* (0.8%).

The overall percent composition by number of crustaceans in lionfish stomach contents was 25.6%, with Decapoda the most frequent prey (93%) followed by Stomatopoda (4.6%) and Euphausiacea (2.4%). Of the 43 crustacean prey sequences, we identified 20 different taxa of which twelve were decapods. Only four showed more than a 90% similarity to reference sequences on BOLD, while the remainder showed similarities between 79 and 89% (Table 4). Three crustacean orders were identified: Euphausiacea with only one species, *Euphausia americana*; Stomatopoda with two samples that apparently belong to Gonodactylidae and Pseudosquillidae, and the remaining matches were all to Decapoda Among the latter, one specimen matched the genus *Synalpheus*, another matched to Hippolytidae and two groups, of three and seven specimens respectively, matched to two clades within Palemonidae. All remaining taxa could not be resolved to a finer level beyond Decapoda (Figure 2).

## Discussion

### Adults

It is possible to distinguish nine species of *Pterois*, including *P. miles* and *P. volitans* from the reference sequences of COI mtDNA in the BOLD database. All of our specimens matched with *Pterois volitans* and the low divergence values among them are consistent with a recent invasion from a small number of specimens. Our results supports the idea that *P. volitans* is the only species which has spread into the Caribbean, including the Mexican region [4], [9], [10], [22]–[24].

### Prey Composition

In the Mexican Caribbean the lionfish (*Pterois volitans*) feeds on a wide diversity of prey, primarily reef-fish species and secondarily crustaceans. These results are concordant with the findings for prey composition of lionfish in the Bahamas [3], [22], [25].

Our values of %N in fishes and crustaceans are similar to those reported by Morris and Adkins in the Bahamas, who found that fishes comprised 71% of the prey items and crustaceans comprised 28.5% [3].

Seven of the identified species constitute range extensions into this area: *Apogon mosavi*, *Coryphopterus venezuelae*, *C. thrix*, *C. tortugae, Lythrypnus minimus*, *Starksia langi* and *S. ocellata*. The first five species are listed for the western and eastern Caribbean even in Belize [26]–[28], so their presence in this region is expected. The lionfish whose stomach contents included *C. tortugae* were collected from Banco Chinchorro and Xcalak. Recently we collected two adults of this specie in the same locality confirming the presence of this species here. *Coryphopterus venezuelae* were detected in three lionfish stomachs from Xcalak. Vásquez Yeomans (Pers. comm.) collected a larva of this species in 2006 in East Cayo Centro, Chinchorro, confirming the presence of this fish in this area. Our six specimens of *Lythrypnus* matched in BOLD with *L. minimus*, one adult from Dominica (LIDMA 726-11) identified by Benjamin Victor (Pers. comm.) and 62 more *Lythrypnus* unidentified sequences. *S. langi* was described recently and is the Belizean species for the species complex of *S. sluiteri* [29] therefore their presence in the Mexican Caribbean is also expected. Finally, *Starksia ocellata* is part of a species complex, named *S. occidentalis* in the Caribbean and the Western Caribbean [30]. This species is a representative with a known range from North Carolina to Florida and the northern Gulf of Mexico. In Mexico there is only a single report in the literature, from Isla Contoy, but there is no voucher specimen. [31].

In the list of prey species, there are five fishes economically important in local markets: *Haemulon flavolineatum* (Chak-chi or French grunt), *Scarus iseri* (loro listado or striped parrotfish), *Sparisoma aurofrenatum* (loro manchado or red band parrotfish), *S. viride* (loro brilloso or stoplight parrotfish) and *Cephalopholis cruentata* (cabrilla, cherna enjambre or graysby). Although these species have not high value in the markets, they are an important source of food for local people.

The yellowhead wrasse, *Halichoeres garnoti* was the most frequent species in the analyzed stomachs, no doubt reflecting its common occurrence in the region [31]. In contrast, *Coryphopterus hyalinus* and *Gramma loreto* have been reported as the most frequent prey of lionfish in the Bahamas, likely reflecting habitat differences in the two locations. [3].

The barcoding of prey species revealed 16 specimens of *Pterois volitans*, although the majority of the samples showed a high degree of digestion (incomplete skeletons with little tissue). Nevertheless, we found one specimen (MXIV868) almost completely intact and morphologically identifiable as *Pterois*. This is the first confirmation of cannibalism among invasive lionfish, a phenomenon that had been previously suggested as likely but with an absence of evidence [22]. The lionfish specimens found in the stomachs were small the intact specimen measured 25 mm SL indicating a preference for juveniles (Figure 3).

Only one specimen in the prey list did not match to any species in the BOLD or GenBank databases. It could be identified to the genus *Astrapogon.* This genus is represented in the Caribbean by three species, all are sequenced in BOLD: *A. punticulatus* was found in the prey samples in this study and the other species, *A. stellatus* and *A. alutus* did not match our sequence, raising the possibility of a cryptic species of *Astrapogon* in the region.

Prior studies in the Bahamas recorded 50 species of fishes in the diet of lionfish based on morphological examination of stomach contents as well as field observations [3], [22], [23]. We found 31 of those species and 17 not previously recorded. Considering the sampling effort for morphological and behavioral analyses, it is evident that DNA barcoding is a more efficient technique, limited for the present only by the incompleteness of the reference databases.

Among crustaceans, the only euphausiid identified was *Euphausia americana* (CRU124.1) from a fish collected in Mahahual (Figure 2). This species has been reported from Xcalak to north of Cozumel Island [32], [33].Two specimens were identified as stomatopods. The sample CRU198 from Playa del Carmen, matched (95% similarity) near the species *Pseudosquilla ciliate*, but with more than 3% divergence, the specimen was considered *Pseudosquilla* sp. The species reported in the Mexican Caribbean are *P. ciliate* [34], [35] and *P. oculata* (IBUNAM:CNCR: CR10740) in the database of the National Collection of Crustaceans from the National Autonomous University of Mexico (UNAM, http://test.unibio.unam.mx), therefore it is possible that this specimen represents *P. oculata*. The other stomatopod (CRU238 from Contoy Island), matched 87.2% nearest to the Gonodactylidae. There are three species reported in the literature of this family in Quintana Roo [34], [35] and the National Collection of Crustaceans in UNAM, *Neogonodactylus bredini* and *N. oerstedii* are in the BOLD database but do not match this sequence, therefore the specimen may represent the third species *N. spinulosus.*

The most frequent crustacean order preyed on by lionfish was decapods, comprising 95% of the crustaceans. Most of the decapods sequences did not match closely to sequences in the reference databases (Table 3), in which case we applied “strict criteria” [36]. Only one sample (CRU 118) was 93% similar to *Thor amboinensis*, and thus considered *Thor* sp. Four species of this genus have been reported in the Mexican Caribbean: *T. amboinensis*, *T. dobkini, T. floridanus and T. manningi* [34], [37], [38]. The sample CRU120 was assigned to the snapping shrimp genus *Synalpheus* (with 99% of similarity), in the Mexican Caribbean there are six species: *Synalpheus fritzmuelleri, S. hemphilli, S. longicarpus*, *S. minus*, *S. townsendi* and *S. apioceros* [35], [39]. There are 19 genera of snapping shrimps in BOLD, none of which matched with our specimen. Two sets of sequences matched to Palemonidae with 88% similarity (CRU136, 138 and 140 and CRU 107, 153, 155, 202,101,141,213). Little is known of the palaemonid fauna of this region and about 32 species of these shrimps have been reported from the shallow waters from Quintana Roo [38].

Crustaceans are an important component of stomach contents studied in most marine fishes, but their identification using morphology is difficult. For example, from 264 crustaceans found in lionfish stomachs from the Bahamas, 246 could not be identified [3]. In contrast, we could identify our 45 crustacean samples to at least the order level. Species level identifications are usually not feasible because of the present incomplete state of the reference databases.

In this study, the methodology yielded 51% efficiency for sequencing. However, most specimens of lionfish were placed into ethanol, with no injection into the viscera or thick muscle. Then, tissues of stomach contents were subsampled one year later, for pcr amplification. In contrast, from 35 tissues taken directly from fresh stomach contents, 29 of them gave good quality sequences, i.e. 83% efficiency, indicating the importance of the fixation process for the samples.

Our results suggest that lionfish are mostly opportunistic predators, eating any prey of appropriate size, consistent with findings from the native range in the Indo-Pacific [40], [25] and including cannibalistic predation on smaller conspecifics as well.

## Materials and Methods

For determination of the *Pterois* species, a small piece (about 1–3 mm^3^) of muscle was removed from 30 specimens collected from Cozumel (25), Xcalak (2), Puerto Morelos (2) and Playa del Carmen (1) and placed in 100% ethanol. To avoid DNA contamination, all tools were flame sterilized before sampling each specimen. The remainder of each fish was retained as a reference voucher in the Fish Collection of El Colegio de la Frontera Sur, Chetumal Unit (ECOCHP).

For the stomach contents analysis, we extracted the stomach from 122 lionfish from whole specimens previously fixed in alcohol, from Banco Chinchorro, Cozumel, Isla Contoy, Isla Mujeres, Puerto Morelos, Playa del Carmen, Xcalak. In case of Mahahual, the digestive tract from 35 fresh lionfish were dissected and placed in 96% ethanol and kept on ice. In total 157 stomachs were analyzed (Table 2). The specimens were collected by personnel from Secretaría del Medio Ambiente y Recursos Naturales (SEMARNAT, Mexico) working in protected areas or volunteers. Collecting methods varied from hand nets, harpoons to plastic bags.

From all stomach contents 1 mm^3^ tissue plugs were extracted from all recognizable material as a prey item under a binocular microscope, after that, the tissue was cleaned with alcohol to avoid contamination from other material.

To extract DNA, the plugs were placed in vertebrate lysis buffer with Proteinase K and digested overnight at 56°C. Genomic DNA was subsequently extracted using a membrane-based approach on the Biomek FX© liquid handling station and AcroPrep 96,1 mL filter plates with 1.0 µM PALL glass fiber media [41]. A 652–658 bp segment of COI was amplified using different fish primers: FishF1, FishR1, FishF2, FishR2 (Ward et al. 2005) or a M13-tailed fish primer cocktail [42].

The 12.5 µL PCR reaction mixes included 6.25 µL of 10% trehalose, 2 µL of ultrapure water, 1.25 µL of 10× PCR buffer, 0.625 µL of MgCl2 (50 mM), 0.125 µL of each primer (0.01 mM), 0.0625 µL of dNTP mix (10 mM), 0.625 µL of Taq polymerase (New England Biolabs or Invitrogen), and 2.0 µL of DNA template. Amplification protocols followed those described in earlier publications [43]. PCR products were visualized on pre-cast agarose gels (E-Gels©, Invitrogen) and the positives, represented by a band were selected for sequencing.

Products were labelled by using the BigDye© Terminator v.3.1 Cycle Sequencing Kit (Applied Biosystems, Inc.) as described [43] and sequenced bidirectionally using an ABI 3730 capillary sequencer following manufacturer’s instructions. Sequence data, electropherograms, trace files, primer details, photographs and collection localities for specimens are available within the project MXLionfish in BOLD (http://www.barcodinglife.org). Sequencing protocols were carried out at the Canadian Centre for DNA Barcoding using standard protocols [43]. Sequences were aligned using SEQSCAPE v.2.1.1 software (Applied Biosystems, Inc.). All COI sequences have also been deposited in GenBank (http://www.ncbi.nlm.nih.gov/, See Table S1 for accession numbers).

The sequences obtained were submitted and identified with the ID engine provided in the Barcode of Life Database (BOLD; www.boldsystems.org) to establish whenever possible the identification of the ingested material. Sequence divergences were calculated using the tools provided by BOLD, the Kimura two parameter (K2P) distance model [44]. Neighbour–joining (NJ) trees based on K2P distances were created to provide a graphic representation of the patterning of divergence between species [45] and a simplified tree was constructed using the MEGA 3 software [46]. The criteria to assign identification to a specie level, was based on less than 3% divergence between the unknown and the reference sequence.

When a sequence match was not found in the DNA barcode reference library, we applied the method for visualization of two trees and based our taxonomical assignment following the strict criteria proposed, and consist in nesting the “unknown” within a clade comprising of members of a single taxon. This criterion was used previously only with moths and 75% of the queries were correctly assigned to genus [36].

## Supporting Information

Table S1 **accession codes from specimens of the public database in BOLD and GenBank (XLS).**
(XLS)

## References

[1] Whitfield PE, Gardner T, Vives SP, Gilligan MR, Courtenay WR (2002). Biological invasion of the Indo-Pacific lionfish *Pterois volitans* along the Atlantic coast of North America.. Marine Ecology-Progress Series.

[2] Schofield PJ (2009). Geographic extent and chronology of the invasion of non-native lionfish (*Pterois volitans* (Linnaeus 1758) and *P. miles* (Bennett 1828)) in the western north Atlantic and Caribbean Sea.. Aquatic Invasions.

[3] Morris JA, Akins JL (2009). Feeding ecology of invasive lionfish *(Pterois volitans*) in the Bahamian archipelago.. Environmental Biology of Fishes.

[4] Hamner RM, Freshwater DW, Whitfield PE (2007). Mitochondrial cytochrome b analysis reveals two invasive lionfish species with strong founder effects in the western Atlantic.. Journal of Fish Biology.

[5] Kochzius M, Soller R, Khalaf MA, Blohm D (2003). Molecular phylogeny of the lionfish genera *Dendrochirus* and *Pterois* (Scorpaenidae, Pteroinae) based on mitochondrial DNA sequences.. Molecular Phylogenetics and Evolution.

[6] Barbour AB, Montgomery ML, Adamson AA, Diaz-Ferguson E, Silliman BR (2010). Mangrove use by the invasive lionfish *Pterois volitans*.. Marine Ecology-Progress Series.

[7] Maljkovic A, Van Leeuwen TE, Cove SN (2008). Predation on the invasive red lionfish, *Pterois volitans* (Pisces: Scorpaenidae), by native groupers in the Bahamas.. Coral Reefs.

[8] Freshwater D, Hamner RM, Parham S, Wilbur AE (2009). Molecular Evidence That the Lionfishes *Pterois Miles* and *Pterois Volitans* Are Distinct Species.. Journal of the North Carolina Academy of Science.

[9] Aguilar-Perera A, Tuz-Sulub A (2010). Non-native, invasive red lionfish (*Pterois volitans* [Linnaeus, 1758]: Scorpaenidae), is first recorded in the southern Gulf of Mexico, off the northern Yucatan Peninsula, Mexico.. Aquatic Invasions.

[10] Vásquez-Yeomans L, Carrillo L, Morales S, Malca E, Morris JA (2011). First larval record of *Pterois volitans* (Pisces: Scorpaenidae) collected from the ichthyoplankton in the Atlantic.. Biological Invasions.

[11] Ruiz-Carus R, Matheson RE, Roberts DE, Whitfield PE (2006). The western Pacific red lionfish, *Pterois volitans* (Scorpaenidae), in Florida: Evidence for reproduction and parasitism in the first exotic marine fish established in state waters.. Biological Conservation.

[12] Ward RD, Zemlak TS, Innes BH, Last PR, Hebert PDN (2005). DNA barcoding Australia’s fish species.. Philosophical Transactions of the Royal Society B-Biological Sciences.

[13] Valdez-Moreno M, Vásquez-Yeomans L, Elías-Gutiérrez M, Ivanova NV, Hebert PDN (2010). Using DNA barcodes to connect adults and early life stages of marine fishes from the Yucatan Peninsula, Mexico: potential in fisheries management.. Marine and Freshwater Research.

[14] Armstrong KF, Ball SL (2005). DNA barcodes for biosecurity: invasive species identification.. Philosophical Transactions of the Royal Society B-Biological Sciences.

[15] Dewaard JR, Landry JF, Schmidt BC, Derhousoff J, Mclean JA (2009). In the dark in a large urban park: DNA barcodes illuminate cryptic and introduced moth species.. Biodiversity and Conservation.

[16] Elías-Gutiérrez M, Martínez-Jerónimo F, Ivanova NV, Valdez-Moreno M (2008). DNA barcodes for Cladocera and Copepoda from Mexico and Guatemala, highlights and new discoveries..

[17] Zeale MR, Butlin RK, Barker GL, Lees DC, Jones G (2011). Taxon-specific PCR for DNA barcoding arthropod prey in bat faeces.. Molecular Ecology Resources.

[18] Budarf A, Burfeind D, Loh W, Tibbetts I (2011). Identification of seagrasses in the gut of a marine herbivorous fish using DNA barcoding and visual inspection techniques.. Journal of Fish Biology.

[19] Carreon-Martínez L, Johnson T, Ludsin S, Heath D (2011). Utilization of stomach content DNA to determine diet diversity in piscivorous fishes.. Journal of Fish Biology.

[20] Meusnier I, Singer G, Landry JF, Hickey DA, Hebert P (2008). A universal DNA mini-barcode for biodiversity analysis..

[21] Hajibabaei M, Smith MA, Janzen DH, Rodriguez JJ, Whitfield JB (2006). A minimalist barcode can identify a specimen whose DNA is degraded.. Molecular Ecology Notes.

[22] Albins MA, Hixon MA (2008). Invasive Indo-Pacific lionfish *Pterois volitans* reduce recruitment of Atlantic coral-reef fishes.. Marine Ecology-Progress Series.

[23] Côté IM, Maljkovic A (2010). Predation rates of Indo-Pacific lionfish on Bahamian coral reefs.. Marine Ecology-Progress Series.

[24] Arias-González EJ, González-Gandara C, Cabrera JL, Christensen V (2011). Predicted impact of the invasive lionfish *Pterois volitans* on the food web of a Caribbean coral reef.. Environmental Research.

[25] Fishelson L (1997). Experiments and observations on food consumption, growth and starvation in *Dendrochirus brachypterus* and *Pterois volitans* (Pteroinae, Scorpaenidae).. Environmental Biology of Fishes.

[26] Floeter SR, Rocha LA, Robertson DR, Joyeux JC, Smith-Vaniz WF (2008). Atlantic reef fish biogeography and evolution.. Journal of Biogeography.

[27] Froese R, Pauly D (2012). FishBase. World Wide Web electronic publication.. http://www.fishbase.org.

[28] Lavett SC, Tyler JC, Davis PW, Jones SR, Smith GD (2003). Fishes of the Pelican Cays, Belice.. Atoll Research Bulletin.

[29] Baldwin CC, Castillo CI, Weigt LA, Victor BC (2011). Seven new species within western Atlantic *Starksia atlantica*, *S. lepicoelia*, and *S. sluiteri* (Teleostei, Labrisomidae), with comments on congruence of DNA barcodes and species, Zookeys.

[30] Greenfield DW (1979). A Review of the Western Atlantic *Starksia ocellata*-Complex (Pisces:Clinidae) with the description of Two New Species and Proposal of Superspecies Status.. Fieldiana Zoology.

[31] Schmitter-Soto JJ, Vásquez-Yeomans L, Caballero-Vázquez J (2000). Lista de peces marinos del Caribe mexicano.. Anales del Instituto de Biología Universidad Nacional Autónoma de México.

[32] Castellanos-Osorio I (1998). Distribución y abundancia de los eufáusidos del estrato superficial del Mar Caribe mexicano.. Caribbean Marine Studies.

[33] Castellanos-Osorio I, Gasca R (2002). Eufáusidos (Crustacea: Malacostraca) del centro y sur del Mar Caribe mexicano.. Revista de Biología Tropical.

[34] Markhan JC, Donath-Hernández FE, Villalobos-Hiriart JL, Cantú Díaz-Barriga A (1990). Notes on the shallow-water marine Crustacea of the Caribbean coast of Quintana Roo, Mexico.. Anales del Instituto de Biología, Universidad Nacional Autónoma de México.

[35] García-Madrigal MS, Campos-Vázquez C, González NE (2002). Sección de crustáceos de la colección de referencia de bentos costero de ECOSUR.. Universidad y Ciencia.

[36] Wilson JJ, Rougerie R, Schonfeld J, Janzen DH, Halwachs W (2011). When species matches are unavailable are DNA barcodes correctly assigned to higher taxa? An assessment using sphingid moths.. BMC Ecology.

[37] Román-Contreras R, Martínez-Mayén M (2009). Shallow water hippolytid shrimps (Crustacea: Decapoda: Caridea) from the Mexican Caribbean coast.. Hidrobiológica.

[38] Román-Contreras R, Martínez-Mayén M (2010). Palaemonidae (Crustacea: Decapoda: Caridea) from the shallow waters from Quintana Roo, Mexican Caribbean coast.. Revista Mexicana de Biodiversidad.

[39] Román-Contreras R, Martínez-Mayén M (2010). Notes on some alpheid shrimps (Decapoda: Caridea) of Thalassia testudinum meadows, from the Central-Southern Mexican Caribbean.. Hidrobiológica.

[40] Whitfield PE, Hare JA, David AW, Harter SL, Muñoz RC (2007). Abundance estimates of the Indo-Pacific lionfish Pterois volitans/miles complex in the Western North Atlantic.. Biological Invasions.

[41] Ivanova NV, De Waard JR, Hebert PDN (2006). An inexpensive, automation-friendly protocol for recovering high-quality DNA.. Molecular Ecology Notes.

[42] Ivanova NV, Zemlak TS, Hanner RH, Hebert PDN (2007). Universal primer cocktails for fish DNA barcoding.. Molecular Ecology Notes.

[43] Hajibabaei M, DeWaard JR, Ivanova NV, Ratnasingham S, Dooh R (2005). Critical factors for assembling a high volume of DNA barcodes.. Philosophical Transactions of the Royal Society of London Series B-Biological Sciences.

[44] Kimura M (1980). A simple method for estimating evolutionary rates of base substitutions through comparative studies of nucleotide sequences.. Journal of Molecular Evolution.

[45] Saitou N, Nei M (1987). The neighbor-joining method: A new method for recosntructing phylogenetic trees.. Molecular Biology and Evolution.

[46] Kumar S, Tamura K, Masatoshi N (2004). MEGA3: Integrated Software for Molecular Evolutionary Genetics Analysis and Sequence Alignment.. Briefings in Bioinformatics.

